# Transient Activation of GABA_B_ Receptors Suppresses SK Channel Currents in Substantia Nigra Pars Compacta Dopaminergic Neurons

**DOI:** 10.1371/journal.pone.0169044

**Published:** 2016-12-30

**Authors:** Chad M. Estep, Daniel J. Galtieri, Enrico Zampese, Joshua A. Goldberg, Lars Brichta, Paul Greengard, D. James Surmeier

**Affiliations:** 1 Department of Physiology, Feinberg School of Medicine, Northwestern University, Chicago, IL, United States of America; 2 Department of Medical Neurobiology, Institute of Medical Research Israel-Canada, Faculty of Medicine, The Hebrew University of Jerusalem, Jerusalem, Israel; 3 Laboratory of Molecular and Cellular Neuroscience, Rockefeller University, New York, NY, United States of America; Indiana University School of Medicine, UNITED STATES

## Abstract

Dopaminergic (DA) neurons in the substantia nigra pars compacta (SNc) are richly innervated by GABAergic neurons. The postsynaptic effects of GABA on SNc DA neurons are mediated by a mixture of GABA_A_ and GABA_B_ receptors. Although activation of GABA_A_ receptors inhibits spike generation, the consequences of GABA_B_ receptor activation are less well characterized. To help fill this gap, perforated patch recordings were made from young adult mouse SNc DA neurons. Sustained stimulation of GABA_B_ receptors hyperpolarized SNc DA neurons, as previously described. However, transient stimulation of GABA_B_ receptors by optical uncaging of GABA did not; rather, it reduced the opening of small-conductance, calcium-activated K^+^ (SK) channels and increased the irregularity of spiking. This modulation was attributable to inhibition of adenylyl cyclase and protein kinase A. Thus, because suppression of SK channel activity increases the probability of burst spiking, transient co-activation of GABA_A_ and GABA_B_ receptors could promote a pause-burst pattern of spiking.

## Introduction

SNc DA neurons play an important role in goal directed movement [1–3] and reward-based learning [4–7]. As a consequence, their electrophysiological properties have been intensively studied [8–12]. Much of this effort has been focused on defining the role of synaptic connections in determining the types of activity patterns seen *in vivo* [13–18].

While a number of studies have explored the excitatory glutamatergic regulation of SNc dopaminergic spiking [14,19–21], far less attention has been paid to GABAergic synapses [18,22,23] despite roughly 70% of the synapses on SNc dopaminergic neurons being GABAergic [24,25]. Like most neurons in the brain, the effects of GABA on SNc dopaminergic neurons are mediated by ionotropic GABA_A_ receptors and G-protein coupled GABA_B_ receptors. In spite of the fact that the GABA_A_ receptor reversal potential is relatively depolarized [26] in SNc DA neurons, their activation clearly slows pacemaking [22,27]. However, the effects of synaptically released GABA on postsynaptic GABA_B_ receptors has been more difficult to ascertain [14,28,29]. What is known is that stimulation of GABA_B_ receptors with exogenous application of agonists leads to activation of K_ir_3 K^+^ channels, hyperpolarizing SNc dopaminergic neurons [30–32].

Although they provide a framework for understanding the effects of GABA on SNc dopaminergic neurons *in vivo*, these experiments have two limitations. First, the time course of receptor activation is much more prolonged than that expected to occur during a phasic burst of activity at GABAergic synapses. Second, much of the work has been conducted with a recording configuration that disrupts the oscillations in cytosolic Ca^2+^ concentration that accompany pacemaking [33,34]. As intracellular Ca^2+^ is a potent modulator of both plasma membrane ion channels and signaling pathways coupled to G-protein coupled receptors, this recording configuration might distort normal cellular responses.

To overcome these limitations, two experimental steps were taken. First, SNc dopaminergic neurons were recorded using the perforated patch technique with gramicidin D. This method only allows monovalent cations to pass between the cell and the electrode [35]. Second, GABA was applied transiently by optically releasing it from a chemical cage [36]. These experiments revealed that transient GABA_B_ receptor stimulation preferentially suppressed SK K^+^ channel currents by inhibiting constitutively active adenylyl cyclase and down-regulating protein kinase A activity. This suppression of SK K^+^ channel currents induced a seconds long period of irregularity in the spiking of SNc dopaminergic neurons. Moreover, given previous work linking SK channels and burst spiking in SNc dopaminergic neurons, GABA_B_ receptor signaling could promote burst spiking after a GABA_A_ receptor induced pause.

## Materials and Methods

### Mice and brain slice preparation

All experiments were performed in accordance with the Northwestern University Animal Care and Use Committee and NIH guidelines. For electrophysiology experiments, male and female P30-45 C57Bl/6 mice were anesthetized with a ketamine/xylazine mixture and then intracardially perfused with ~4°C, high-sucrose, high-Mg^2+^ slicing aCSF containing (in mM) 50 NaCl, 2.5 KCl, 25 NaHCO_3_, 1.25 NaH_2_PO_4_, 1 CaCl_2_, 10 MgCl_2_, 25 glucose, pH 7.3 (~310 mOsm/L) just prior to decapitation and brain extraction. Coronal brain slices containing the SNc were cut at 300 μm with a Leica VT1200 S vibratome in slicing aCSF, and placed in a RT recovery solution containing (in mM) 82.5 NaCl, 2.5 KCl, 25 NaHCO_3_, 1.25 NaH_2_PO_4_, 1.5 CaCl_2_, 5.5 MgCl_2_, 25 glucose, pH 7.3 (~310 mOsm/L) until recording. All solutions were oxygenated with a mixture of 95% O2/5% CO2.

### Electrophysiology

For patch-clamp recordings, slices were washed with aCSF containing (in mM) 125 NaCl, 2.5 KCl, 25 NaHCO_3_, 1.25 NaH_2_PO_4_, 2 CaCl_2_, 1 MgCl_2_, 25 glucose, pH 7.3 (~310 mOsm/L) and then placed in a recording chamber perfused continuously at ~2 mL/min with oxygenated aCSF kept at ~35–37°C and allowed to acclimate for a minimum of 15 minutes before recording. Cells were visualized on an Olympus BX51 upright microscope outfitted with IR DIC optics with a 40x water-immersion objective (NA 0.8). Patch pipettes were pulled from thick-walled borosilicate glass with a Sutter P-1000 puller. Pipettes had a tip resistance between 1.5–3 MΩ when filled with internal solution. To prevent dialysis with the patch electrode all experiments, with the exception of those measuring Ca^2+^ channel currents, were performed using the gramicidin-D perforated patch configuration. Pipettes used for perforated patch recordings were first front-filled with a solution containing (in mM) 126 KMeSO_4_, 14 KCl, 10 HEPES, 1 EGTA, 0.5 CaCl_2_, 3 MgCl_2_, pH 7.3 (~280 mOsm/L), and then back-filled with the same solution containing ~20 μg/mL gramicidin-D. Calcium current recordings were conducted in a whole-cell patch-clamp configuration with an internal solution containing (in mM) 120 CsMeSO_3_, 15 CsCl_2_, 10 HEPES, 0.2 EGTA, 3 ATP-Mg, 0.3 GTP-Na, 10 TEA-Cl, pH 7.3 (~280 mOsm/L) and an external solution containing (in mM) 145 TEA-Cl, 2.5 CsCl_2_, 10 HEPES, 2 CaCl_2_, 1 MgCl_2_, 25 glucose, pH 7.3 (~310 mOsm/L), in addition to NBQX, (R)-CPP, SR95531, TTX, isradipine, and omega-conotoxin-GIVA at concentrations denoted in the pharmacology methods. Total calcium current was antagonized with cadmium [500 μM] and subtracted from analyzed traces. All recordings were digitized at 10 kHz and low-pass filtered with a 1 kHz cutoff Bessel.

### GABA photolysis

RuBi-GABA [5μM] for uncaging experiments was purchased from Abcam (Cambridge, UK) and stock solutions were made and diluted within one week of use. Wide-field, 50 ms, 500 ms, and 1 min pulses from a CooLED (Andover, UK) pe-100 LED centered at 470nm (100% power) were used to uncage RuBi-GABA around the soma of the recorded cell.

### Pharmacology

Electrophysiology and imaging experiments used a variety of pharmacology. Stocks of drugs were made according to manufacturer instructions and diluted ~1000x just prior to experiments. Experimental drugs were all purchased from R&D Systems (Minneapolis, MN) or Santa-Cruz Biotechnology (Dallas, TX) and concentrations were as follows: NBQX [5 μM], (R)-CPP [5 μM], SR95531 [5–25 μM], CGP55845 [2 μM], baclofen [5 μM], apamin [300 pM-200 nM], tetrodotoxin [1 mM], isradipine [20 μM], omeaga-conotoxin-GIVA [10 nM], H89 [10 μM], Rp-8-CPT-cAMPS [100 μM], mibefradil [10 μM], 8-bromo-cAMP [1 μM], CPA [10 μM], dantrolene [10 μM]. All experimental drugs were made to their suggested stock concentrations and stored according to their instructions. Drugs were diluted to their final concentrations just prior to experiments.

### Endoplasmic reticulum(ER) imaging

For ER imaging experiments, P17-P21 C57Bl/6 mice were stereotaxically injected with 350 nl AAV9 packaged with TH-G-CEPIA1er construct (1.5e^13^ vp/ml, Virovek) in their right midbrain region (stereotaxic coordinates: DV: 4.5; ML: 1.3; RC: 3.1, adjusted for the size of each mouse according to the distance between Bregma and Lambda) and sacrificed at least 10 days after the injection (P26-P36) and coronal midbrain slices (220 μm) were collected and used for experiments.

### 2-Photon Laser Scanning Microscopy (2PLSM)

Brain slices were placed on an upright microscope heated chamber (~33°C) and perfused at a constant rate of ~2 mL/min with aCSF and allowed to stabilize for at least 15 mins before starting the acquisitions. Optical imaging of G-CEPIA1er signals were acquired using a 920-nm excitation beam (80-MHz pulse repetition frequency and ~250-fs pulse duration), in a fixed plane of focus with a pixel size between 0.18 and 0.21 μm and a 12-μs pixel dwell time. The G-CEPIA1er fluorescence was detected by a GaSP PMT and a Dodt contrast-detector system that provided a bright-field transmission image (Prairie Technologies). Images were acquired with an Olympus LUMPFL 60×/1.0 NA water-dipping objective lens. One or two cell bodies were defined as a region of interest (ROI) for each experiment. Twenty frames of the G-CEPIA1er signal were collected in one optical plane at a rate of 3–4 frames per second. Acquisitions were taken every 10 mins for the baseline and the recovery phases, more frequently (1–5 mins) during stimulations.

### Electrophysiology and imaging analysis

Data were analyzed using a custom written python analysis package (Neurphys; https://github.com/surmeierlab/neurphys) and MatLab scripts. Figures were created with Matplotlib [37] and Adobe Illustrator. All box plots presented as median, first and third quartiles, and whiskers at 10th and 90th percentiles. Data outside that range is represented as individual points.

### TRAP tissue preparation

All experiments were approved by the Rockefeller University Institutional Animal Care and Use Committee and performed in accordance with the guidelines described in the US National Institutes of Health Guide for the Care and Use of Laboratory Animals. Mice were housed in rooms on a 12 h dark/light cycle at 22°C and maintained with rodent diet (Picolab) and water available ad libitum. 30 four-month-old male hemizygous Dat bacTRAP mice [38] were randomly divided into six groups of five mice. Brains were removed and sectioned using an ice-cold Adult Mouse Brain Slicer with 1 mm coronal slice intervals (Zivic Instruments). From the tissue section containing the midbrain, the SNpc and VTA regions were dissected and separated under a Nikon SMZ645 light microscope using a 10x lens.

### RNA sequencing and analysis

Translated mRNAs were purified as described previously (Heiman et al. Nature Protocols 2014). TRAP samples underwent DNase digestion using the RNase-Free DNase Set (Qiagen) and were subsequently purified with the RNeasy MinElute Cleanup Kit (Qiagen). Eluted RNA samples were analyzed on a 2100 Bioanalyzer (Agilent) using RNA Pico Chips (Agilent) to confirm RNA integrity, followed by the measurement of RNA concentrations with the Quant-iT RiboGreen RNA Assay Kit (Life Technologies). cDNAs were prepared with the Ovation RNA-Seq System V2 kit (NuGEN), using an input of 1 ng RNA. 500 ng cDNA from each sample were fragmented on a Covaris S2 Focused Ultrasonicator using the operating conditions recommended by the manufacturer for a target fragment size of 200 bp. Fragment size was confirmed on a 2100 Bioanalyzer using High Sensitivity DNA Chips (Agilent). Libraries for RNA sequencing were prepared with the TruSeq RNA Sample Preparation v2 kit (Illumina), starting the manufacturer’s low-throughput protocol with the end repair step. The concentration of the RNA-Seq libraries was determined on a 2100 Bioanalyzer using High Sensitivity DNA Chips. Subsequently, two libraries with different adapters were multiplexed for sequencing. After confirming the concentration of the multiplexed samples on a 2100 Bioanalyzer using High Sensitivity DNA Chips, samples were analyzed on an Illumina HiSeq 2000 sequencer using 100 bp single-end sequencing. RNA-Seq reads were mapped to the Mus musculus assembly 10 reference genome using TopHat version 2.0.11. FPKM values for all genes in each sample were calculated with Cufflinks version 2.2.1. To analyze differential gene expression between samples, DESeq version 1.14.0 was used under the standard comparison mode. P values were reported by DESeq, adjusted for multiple testing using the Benjamini-Hochberg procedure.

## Results

### Transient stimulation of GABA_B_ receptors has distinct effects

**Fig 1:**
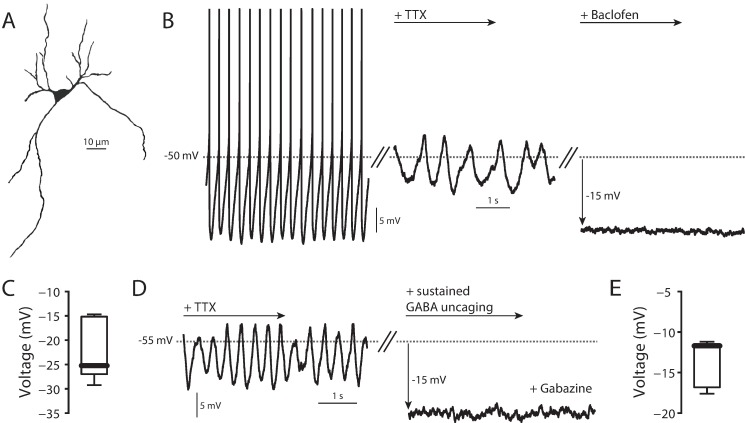
SNc DA neuron physiology. (A) 2P reconstruction of SNc DA neuron. (B) Left, normal pacemaking of a SNc DA neuron. Middle, TTX (1 μM) application uncovered slow oscillatory potential (SOP). Right, 5 μM baclofen application hyperpolarized the cell. (C) Summary of hyperpolarization due to application of 5 μM baclofen (n = 8, median = -25.24 mV). (D) Sustained uncaging of 5 μM RuBi-GABA in the presence of 25 μM gabazine (to block GABA_A_ receptors) hyperpolarized cells in a manner similar to that seen following baclofen application. (E) Summary of hyperpolarization due to sustained 5 μM RuBi-GABA uncaging (n = 7, median = -11.71 mV).

In *ex vivo*, coronal midbrain slices taken from P30-P45 mice, SNc DA neurons (Fig 1A) recorded using the perforated patch configuration exhibited regular, pacemaking activity (1–6 spikes/s) (Fig 1B) [31,39–41]. When the Na_V_1 channel antagonist tetrodotoxin (TTX, 1 μM) was added to the bath, slow oscillatory potentials (SOPs) appeared, which had a dominant frequency slower than that of pacemaking (Fig 1B) [33,34,42,43]. These SOPs were dependent upon opening of Ca_V_1 Ca^2+^ channels, as bath application of dihydropyridines at low micromolar concentrations eliminated them [33,34]. Bath application of the GABA_A_ receptor antagonist gabazine (25 μM) did not alter either basal spiking rate or SOPs, suggesting that there was no tonic GABAergic tone with superfused slices (data not shown). As shown previously, bath application of the GABA_B_ receptor agonist baclofen (5 μM) induced membrane hyperpolarization (Fig 1B). Sustained uncaging of Rubi-GABA (5 μM) in the presence of the GABA_A_ receptor antagonist SR-95531 (gabazine, 25 μM) [44,45] also induced a consistent membrane hyperpolarization (Fig 1D). These effects are in agreement with the previously described coupling of GABA_B_ receptors through G_i/o_ proteins to K_ir_3 K^+^ channels [30,46].

**Fig 2:**
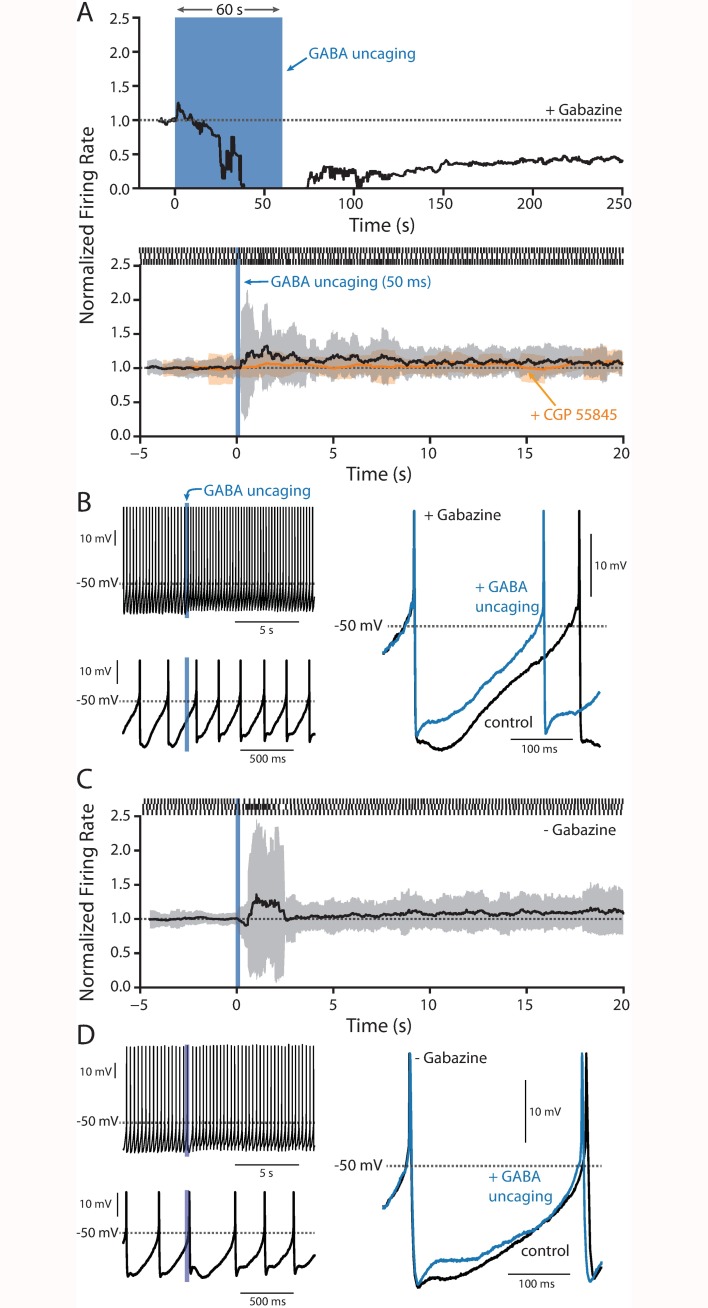
RuBi-GABA uncaging. (A) Top, plot of the normalized firing rate before during and after a 60 s uncaging pulse (blue bar) of 5 μM RuBi-GABA in the presence of 25 μM gabazine (n = 4). Bottom, plot of normalized firing rate (black line) and running standard deviation (grey area) before, during and after a 50 ms uncaging pulse in the presence of 5 μM RuBi-GABA and 25 μM gabazine (n = 12); application of 2 μM CGP 55845 blunted the changes in spiking induced by RuBi-GABA uncaging (orange line, n = 4). Example raster plots are shown at the top of the panel. (B) Left, two different time scales showing action potentials just prior to and after GABA uncaging. Right, overlaid action potentials from just prior to and after GABA uncaging showing a clear reduction in the mAHP. (C-D) As in panel A (bottom) and B, but in the absence of gabazine (n = 9).

To determine if the duration of GABA_B_ receptor activation had an impact on the physiology of SNc neurons, RuBi-GABA (5 μM) was uncaged for varying periods of time using full-field LED illumination in the presence of gabazine (25 μM) to block GABA_A_ receptors, and the effect on pacemaking monitored. A one minute uncaging pulse led to the expected slow hyperpolarization of the membrane and the eventual cessation of firing seen with bath application of baclofen (Fig 2A). The onset of the hyperpolarization was slow, consistent with either a concentration dependence or a kinetically slow coupling mechanism. In contrast, a single 50 ms uncaging pulse caused a small, but consistent increase in discharge rate (Fig 2A) with a more pronounced increase in the irregularity of spiking as measured by interval standard deviation (Fig 2A). Within seconds, spiking returned to its normal rate and regularity. Bath application of the GABA_B_ receptor antagonist CGP 55845 (2 μM) prior to GABA uncaging blocked changes in firing frequency and regularity (Fig 2A). Moreover, omission of the GABA_A_ receptor antagonist gabazine during GABA uncaging did restore a brief delay in the next spike latency attributable to GABA_A_ receptors; however, it did not qualitatively change the subsequent increase in discharge rate and irregularity (Fig 2C and 2D).

Closer inspection of the spikes revealed that GABA uncaging reduced the magnitude of the medium afterhyperpolarization (mAHP) (Fig 2B–2D). The omission of gabazine did not alter the ability of GABA uncaging to reduce the mAHP (Fig 2D). In SNc dopaminergic neurons, the mAHP is dominated by currents through SK K^+^ channels [33,47–49]. These channels modulate both the rate and regularity of spiking [48–52] and have a well-established role in the SOP [33,53].

**Fig 3:**
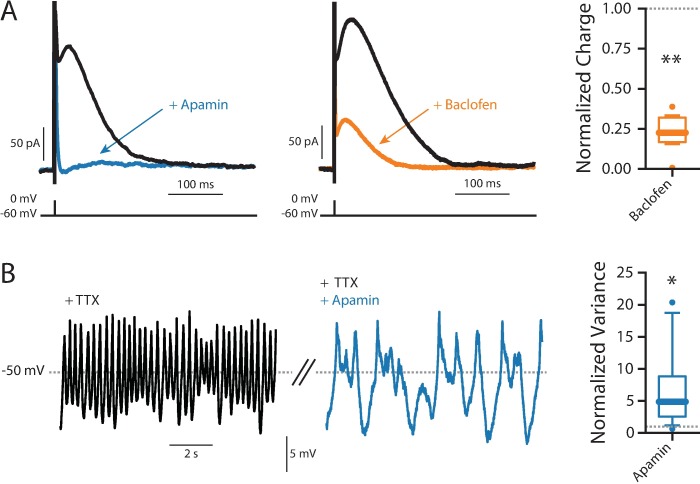
SK channels. (A) Example SK voltage-clamp recordings showing baseline (black), and responses to 200 nM apamin (blue) and 5 μM baclofen (orange). Right, summary of normalized response to baclofen (n = 8, Wilcoxon signed rank test, p = 0.0078). (B) SOPs (black) are greatly slowed and increase in amplitude with exposure to 200 nM apamin (blue). Right, summary of the normalized variance (n = 9, Wilcoxon signed rank test, p = 0.0151).

To directly assess the ability of GABA_B_ receptor signaling to modulate SK channels, a hybrid clamp technique was used. In perforated patch recordings, a brief current pulse was used to evoke a spike and the cell was voltage clamped at -60 mV, allowed SK K^+^ currents to be reliably measured [48]. The SK channel antagonist apamin almost completely eliminated the post-spike current (Fig 3A). Baclofen (5 μM) also dramatically reduced the post-spike current, providing direct evidence of SK channel modulation by GABA_B_ receptors (Fig 3A).

As mentioned above, SK channels have also been implicated in the regulation of the SOP [33,53]. In agreement with previous studies using sharp electrodes [53], in perforated patch recordings apamin (200 nM) caused the SOP to slow and become more irregular (Fig 3B).

### GABA_B_ receptor signaling inhibited adenylyl cyclase

**Fig 4:**
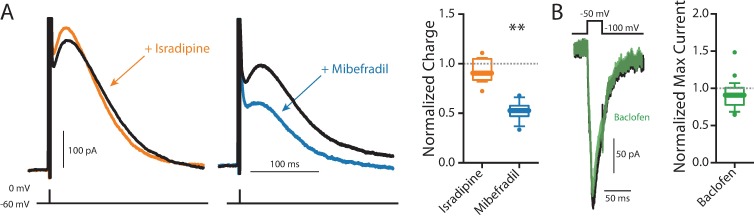
VGCC contribution to SK. (A) Application of 10 μM isradipine (orange) to inhibit Ca_V_1 channels did not reduce total SK charge (n = 8, Wilcoxon signed rank test, p = 0.3828), while inhibiting Ca_V_3 channels with 10 μM mibefradil (blue) inhibited roughly half the charge (n = 8, Wilcoxon signed rank test, p = 0.0078). (B) 5 μM baclofen (green) application did not inhibit T-type calcium current (n = 12, Wilcoxon signed rank test, p = 0.1099).

GABA_B_ receptors couple to G_i/o_ signaling pathways. In addition to activating K_ir_3 K^+^ channels, G_i/o_-protein coupled receptors can inhibit voltage-dependent Ca^2+^ channels [54,55]. This type of modulation could mediate the GABA_B_ receptor suppression of Ca^2+^ activated SK channels. In agreement with previous work [48], an inhibitor of Ca_V_3 (mibefradil, 10 μM) but not Ca_V_1 (isradipine, 10 μM) Ca^2+^ channels decreased SK channel currents measured with the hybrid clamp (Fig 4A). However, there was no measureable effect of baclofen (5 μM) on Ca_V_3 channel currents measured with a voltage step from -100 mV to -50 mV (Fig 4B). Although it is possible that incomplete voltage clamp obscured a modest modulation of these currents by GABA_B_ receptor activation, the more plausible interpretation is that the SK channel modulation was mediated by some other mechanism.

**Fig 5:**
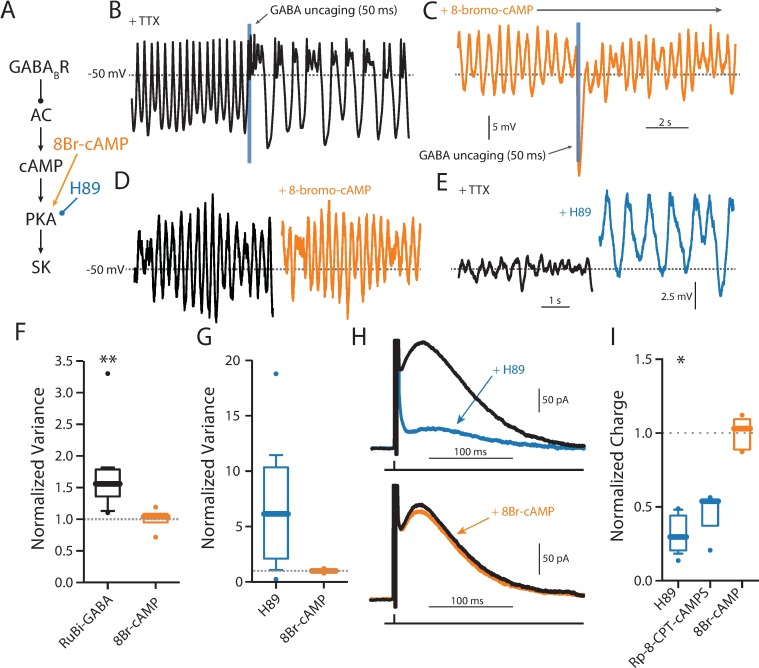
PKA activation prevents GABA_B_ modulation of SK. (A) Schematic diagram showing the hypothesized signaling pathway from GABA_B_ receptor activation to SK channels, and the site of action of 8-bromo-cAMP and H-89 in that pathway. (B) A 50 ms uncaging pulse elicited an immediate and significant change in SOP variance (black, n = 10, Wilcoxon signed rank test, p = 0.002). (C) The same was not seen when cells were incubated in 1 μM 8-bromo-cAMP (orange, n = 4, Wilcoxon signed rank test, p = 0.875). (D) Directly activating PKA with 1 μM 8-bromo-cAMP does not have an effect on SOP variance (n = 4, Wilcoxon signed rank test, p = 0.75). (E) Inhibiting PKA with 10 μM H89 increases SOP variance (n = 6, Wilcoxon signed rank test, p = 0.0938). (F) Summary data for panels A-B. (G) Summary data for panels C-D. (H) Top, inhibiting PKA with 10 μM H89 significantly decreases SK current (n = 6, Wilcoxon signed rank test, p = 0.0313). Bottom, directly activating PKA with 1 μM 8-bromo-cAMP does not have an effect on SK current (n = 5, Wilcoxon signed rank test, p = 1.00). (I) Summary data for PKA modulators from panel H and Rp-8-CPT-cAMPS (n = 3, Wilcoxon signed rank test, p = 0.25).

To narrow the range of potential targets, an attempt was made to determine the signaling elements downstream of GABA_B_ receptors. One of the best described targets of G_i/o_ signaling is adenylyl cyclase (AC) [56,57]. SNc dopaminergic neurons express AC1 isoforms that are stimulated by Ca^2+^ (S1 Table), raising the possibility that this form of AC is constitutively active during pacemaking. To test this hypothesis, the ability of GABA_B_ receptors to modulate the SOP was examined before and after perturbing AC signaling. First, as shown above, a single, brief uncaging of GABA induced a slowing of SOP frequency and an increased irregularity in the oscillation (Fig 5A). Bath application of a membrane permeable analog of cyclic adenosine monophosphate (cAMP) (8-bromo-cAMP, 1 μM) blunted this modulation (Fig 5B and 5C). However, bath application of 8-bromo-cAMP (1 μM) had no discernible effect on SOPs (Fig 5D), consistent with the proposition that AC was constitutively active and GABA_B_ receptors were having their effect by transiently suppressing this activity.

The cAMP generated by AC activates protein kinase A (PKA), leading to phosphorylation of a wide range of substrates. If GABA_B_ receptor signaling was inhibiting AC, it should result in diminished PKA activity. Thus, PKA inhibition should mimic GABA_B_ receptor signaling. To test this idea, the PKA inhibitor H-89 (10 μM) was bath applied. As predicted, H-89 slowed the SOP and increased its irregularity and amplitude (Fig 5E and 5G–5I). To directly test the role of AC signaling in regulating SK channels, the hybrid clamp was used. As expected, 8-bromo-cAMP had no effect on SK channel currents (Fig 5H). However, H-89 dramatically suppressed SK channel currents (Fig 5H), arguing that constitutive AC and PKA activity was critical to maintaining SK activity and that GABA_B_ receptor inhibition of AC could mediate SK channel inhibition. Similar results were seen with another PKA antagonist, Rp-8-CPT-cAMPS (100 μM) (Fig 5I).

### Depletion of intracellular Ca^2+^ stores did not block SK modulation

**Fig 6:**
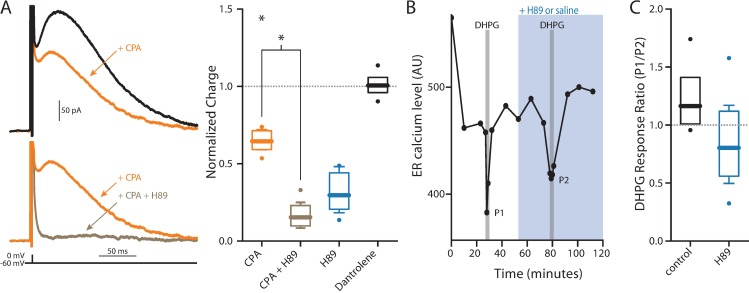
ER Ca^2+^ contribution to SK current. (A) Application of CPA to empty ER Ca^2+^ stores significantly reduced SK current (orange, n = 6, Wilcoxon signed rank test, p = 0.0313) and further application of 10 μM H89 to inhibit PKA activity further significantly reduced SK current (brown, n = 6, Wilcoxon signed rank test, p = 0.0313), but does not reduce it any further than H89 alone (Mann-Whitney U test, p = 0.1320). Inhibiting ryanodine receptors with 10 μM dantrolene did not change SK current (black, n = 4, Wilcoxon signed rank test, p = 1.00). (B) Left, an example experiment showing the effect of DHPG application on ER Ca^2+^ levels. Right, summary of normalized data showing a small, but not significant change in induced ER Ca^2+^ release after application of H89 (n = 4) compared to control (n = 7) recordings (Mann-Whitney U test, p = 0.2303).

SK channels in SNc dopaminergic neurons also are known to be regulated by Ca^2+^ released from intracellular stores controlled by inositol 1,4,5-triphosphate (IP3) receptors [58,59]. PKA phosphorylation of IP3 receptors enhances their opening [60–62]. Thus, one potential way in which PKA might promote SK activation is by enhancing IP3 receptor mobilization of Ca^2+^. Depleting intracellular Ca^2+^ stores by inhibiting the smooth endoplasmic reticulum Ca^2+^ ATPase (SERCA) with cyclopiazonic acid (CPA, 10 μM) reduced SK channel currents measured with the hybrid clamp, in agreement with previous work [63]. However, in the presence of CPA, the PKA inhibitor H-89 (10 μM) continued to suppress SK channel currents (Fig 6A), suggesting that PKA was not constitutively effecting SK channels by enhancing IP3 receptor function.

**Fig 7:**
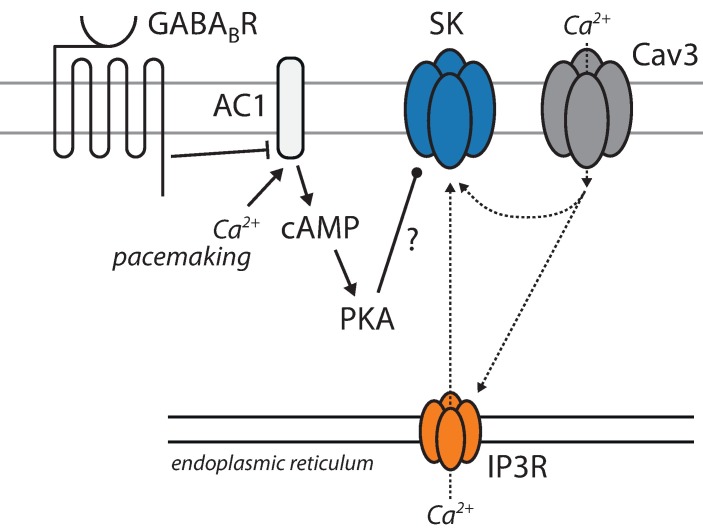
Schematic diagram depicting hypothesized signaling pathways involved in the GABA_B_ receptor-mediated inhibition of SK channels. GABA_B_ receptor inhibition of AC by G_i_ signaling is hypothesized to be responsible for reduced cAMP levels and PKA signaling. The reduction in PKA activity is hypothesized to reduce SK channel opening through mechanism that are independent of either plasma membrane Ca^2+^ channels or release from intracellular stores.

To provide an additional test of this inference, the effects of PKA inhibition on the ability of group 1 metabotropic glutamate receptors (mGluRs) to deplete ER Ca^2+^ stores was assessed. If PKA was constitutively enhancing IP3 receptor function, inhibiting PKA should blunt the ability of mGluRs to activate IP3 receptors and deplete ER stores. To test this prediction, group 1 mGluRs were activated with (*S*)-3,5-dihydroxyphenylglycine (DHPG, 10 μM). DHPG produced a robust lowering of ER Ca^2+^ concentration measured in *ex vivo* brain slices with the genetically encoded ER Ca^2+^ probe CEPIA1er [64]. However, H-89 (10 μM) failed to significantly diminish the DHPG evoked drop in ER Ca^2+^ (Fig 6B), suggesting that PKA does not constitutively enhance IP3R-mediated Ca^2+^ release in SNc dopaminergic neurons. Application of the ryanodine receptor (RyR) antagonist dantrolene (10 μM) had no effect on SK currents, suggesting that RyRs do not contribute to SK channel activation. Taken together, these results argue that PKA is regulating SK channel gating through a mechanism that is independent of ER stores (Fig 7).

## Discussion

Our studies have identified a novel mechanism of GABA_B_ receptor modulation of SNc dopaminergic neuron activity. While confirming that GABA_B_ receptor signaling can activate K_ir_3 K^+^ channels and suppress spiking when stimulation is sustained, our results show that transient activation of GABA_B_ receptors has a very different effect on ongoing pacemaking. Rather than inhibiting spiking, transient activation of GABA_B_ receptors increased spiking rate and irregularity. This effect on spiking was mediated by suppression of SK channel currents. Given the well-established ability of SK channel inhibition to increase the propensity of SNc dopaminergic neurons to spike in bursts [19,51], a natural inference from our studies are that *in vivo* GABA_B_ receptors signaling could create a seconds long window in which subsequent glutamatergic input could induce burst spiking more easily.

### Transient elevation in GABA induced suppression of SK currents

Using bath application of ligands, previous studies of GABA_B_ receptor effects on SNc dopaminergic neurons have repeatedly found that they are capable of activating K_ir_3 K^+^ channels, leading to hyperpolarization and the cessation of ongoing, autonomous pacemaking [30,31]. The ability of G_βγ_ proteins released by stimulation of G_i/o_ coupled receptors to increase the open probability of K_ir_3 K^+^ channels through a membrane delimited signaling pathway has been extensively characterized in both native and heterologous expression systems [65–67]. This is a robust modulation that is resistant to the alterations in intracellular environment brought about by whole cell or sharp electrode intracellular recording techniques. Using the perforated patch technique, which largely preserves the intracellular milieu, including the oscillation in intracellular Ca^2+^ concentration that accompanies pacemaking [34,68], this GABA_B_ receptor modulation also was evident with bath application of GABA_B_ receptor agonists. Thus, there is no apparent negative regulator of K_ir_3 K^+^ channel modulation in SNc neurons that are pacemaking and have normal fluctuations in intracellular Ca^2+^.

However, with brief stimulation of GABA_B_ receptors enabled by optical uncaging of GABA, there was little evidence of K_ir_3 K^+^ channel activation in most cells (Fig 5C). Rather, uncaging GABA evoked a transient increase in discharge rate and a more prolonged period of irregularity in spiking. Both effects were attributable to a suppression in the mAHP generated by SK K^+^ channel currents. This was shown not only by inspection of the voltage-trajectory of the somatic membrane potential but also with use of hybrid clamp techniques that allowed spike generated outward currents to be isolated. In these experiments, the SK K^+^ channel blocker apamin mimicked the effects of GABA_B_ receptor agonists. Apamin also is known to increase the irregularity of the SOP created by blocking Na_V_1 Na^+^ channels in SNc dopaminergic neurons. Again, this effect was mimicked by GABA_B_ receptor agonists.

In contrast the modulation of K_ir_3 channels, the effect of GABA_B_ receptors on SK channels appeared to be mediated by G_αi_ inhibition of adenylyl cyclase. There are several observations consistent with this conclusion. First, mRNA profiling of SNc DA neurons demonstrated they robustly express AC1, a isoform of adenylyl cyclase that is stimulated by Ca^2+^/calmodulin [69,70]. As pacemaking SNc DA neurons have high levels of intracellular Ca^2+^ [34], AC1 should be constitutively activated. Previous work with whole cell recording where this intracellular Ca^2+^ oscillation was disrupted could have missed the GABA_B_ receptor modulation of SK channels because AC1 activity was reduced. Second, bath application of a membrane permeable cAMP analog (bypassing AC1) blunted the GABA_B_ receptor modulation of SK channels. Moreover, as expected if there was constitutive activity of AC1, the cAMP analogue had no effect in the absence of GABA_B_ receptor stimulation. Third, inhibiting PKA, a major target of cAMP signaling, mimicked the effects of GABA_B_ receptor signaling and occluded the effects of GABA_B_ receptor activation. All three of these observations point to a simple signaling model (Fig 7).

What is unresolved is how PKA signaling enhances SK K^+^ channel gating. Neither source of Ca^2+^ involved in SK K^+^ channel gating–Ca_V_3 Ca^2+^ channels and IP3 receptor sensitive, ER Ca^2+^ stores [48,63]–appeared be affected by PKA inhibition. However, it is possible that our assays of PKA modulation of these targets was not sensitive enough. It is also possible that the SK channel itself is a target of PKA. SK2 and SK3 channels are expressed by SNc DA neurons [47,50] and both channels have serine/threonine phosphorylation sites [71,72]. Although these sites have been reported to control membrane trafficking, it is not clear whether they also affect channel gating.

Another unresolved question is why transient uncaging of GABA was effective in triggering modulation of SK channels but not K_ir_3 channels. As the duration of GABA uncaging will affect the concentration of GABA achieved in the extracellular space, one possibility is that there are high and low affinity GABA_B_ receptors that differentially couple to SK and K_ir_3 channels, but there is no evidence for this kind of GABA_B_ receptor heterogeneity. Another possibility is that differential scaffolding of targets in the neighborhood of GABA_B_ receptors is responsible. It is possible that in SNc dopaminergic neurons the density of K_ir_3 channels in the neighborhood of GABA_B_ receptors is low and that G_βγ_ subunits released by receptor binding have to diffuse a substantial distance to interact with them, slowing the modulation. AC1, on the other hand, could be held near GABA_B_ receptors by scaffolding proteins [55], allowing G_i_ subunits to quickly inhibit enzymatic activity.

### Could GABA_B_ receptors contribute to burst spiking?

Based upon previous work identifying K_ir_3 channels as targets of GABA_B_ receptor signaling, it is widely assumed that GABA_B_ receptors inhibit SNc DA neuron spiking much like GABA_A_ receptors. Our results suggest an alternative scenario. *In vivo*, SNc DA neurons often follow a pause in spiking with a period of increased spiking or bursting [16,27]. This pattern of activity, particularly bursting, is thought to have profound effects on target structures like the striatum by transiently elevating extracellular dopamine concentration. This transient elevation has been linked to reward prediction errors as well as the initiation of movement [4,73].

It is widely held that burst spiking in SNc DA neurons is driven by excitatory glutamatergic synaptic activity [14]. Much like apamin [19], the transient suppression of SK K^+^ channel currents by GABA_B_ receptors should make it easier for glutamatergic synapse to drive bursting [19,20,23,74]. Thus, glutamatergic input to SNc DA neurons that temporally lagged a GABAergic volley from striatal spiny projection neurons that engaged GABA_B_ receptors, would be very effective in evoking a burst of spikes. However, if the GABAergic input was maintained for a longer period of time, it could lead to engagement of K_ir_3 K^+^ channels and suppression of responsiveness to glutamatergic inputs.

## Supporting Information

S1 Table AC expression profile of SNc dopaminergic neurons. AC1-10 (n = 6).
(XLSX)

S1 Dataset Figure data. All data from figure boxplots.
(XLSX)
